# Feasibility and Safety of High-Flow Nasal Cannula Use During Dental Treatment: A Pilot Study

**DOI:** 10.3390/dj14040208

**Published:** 2026-04-02

**Authors:** Terumi Ayuse, Kaori Yamaguchi, Takao Ayuse, Stanislav Tatkov

**Affiliations:** 1 Department of Special Care Dentistry, Nagasaki University Hospital, Nagasaki 852-8501, Japan; ttagawa@nagasaki-u.ac.jp (T.A.); yamaguchi.k925@nagasaki-u.ac.jp (K.Y.); 2 Clinical Research Center, Nagasaki University Hospital, Nagasaki 852-8501, Japan; 3 Fisher & Paykel Healthcare Ltd., Auckland 1741, New Zealand; stanislav.tatkov@fphcare.co.nz

**Keywords:** dentistry, high flow nasal cannula, anxiety, swallowing saliva

## Abstract

**Background:** Dental treatment often requires prolonged mouth opening. This may compromise comfort during spontaneous nasal breathing and saliva swallowing, leading to stress or anxiety. A high-flow nasal cannula (HFNC) delivers warmed and humidified air at high flow rates and may improve breathing comfort; however, the feasibility of its routine use during dental treatment has not been established. **Objectives:** The primary objective of this pilot study is to evaluate the feasibility of conducting a definitive clinical trial to investigate the use of a HFNC during dental treatment. The secondary objective is to explore preliminary patient-centered outcomes related to stress and comfort to inform the design of future clinical trials. **Methods:** This single-center, open-label pilot feasibility study will be conducted at Nagasaki University Hospital, with adult patients undergoing routine full-mouth periodontal treatment participating in two treatment sessions, one without a HFNC and one with a HFNC, separated by at least four weeks. The primary feasibility outcomes include recruitment and retention rates, patient tolerance and acceptability of the HFNC, completeness of data collection, and device-related adverse events. The secondary outcomes are exploratory and include physiological stress-related parameters (pulse rate, respiratory rate, autonomic nervous system indices, and electroencephalographic alpha wave activity) and patient-reported comfort assessed using a questionnaire. **Conclusions:** This pilot study was designed to assess the feasibility and safety of HFNC use during full-mouth periodontal treatment and to inform the design of future definitive clinical trials. In particular, the resultant exploratory patient-centered outcomes and preliminary data may be used to guide outcome selection and sample size estimation.

## 1. Introduction

Dental treatment frequently requires patients to maintain a wide-open mouth for prolonged periods while breathing through the nose. This posture can make nasal breathing and swallowing difficult, resulting in discomfort, anxiety, and stress. In some patients, maladaptive breathing patterns, such as hyperventilation or breath-holding, may occur as coping strategies, potentially compromising both patient comfort and procedural safety. Dental anxiety and treatment-related distress remain common clinical problems. Contemporary evidence highlights persistent gaps and heterogeneity in interventions aimed at reducing acute anxiety during dental procedures, underscoring the need for pragmatic, low-burden approaches that can be integrated into routine workflows [1].

High-flow nasal cannula (HFNC) therapy delivers warm and humidified air or oxygen at high flow rates. HFNCs are widely used in medicine, anesthesiology, and critical care. The HFNC is known to generate low levels of positive airway pressure, wash out nasopharyngeal dead space, and reduce breathing during spontaneous respiration [2]. Previous experimental and clinical studies have suggested that the HFNC may facilitate the swallowing of saliva and improve the coordination between breathing and swallowing under spontaneous breathing conditions [3,4,5,6,7]. These physiological effects indicate that the HFNC may be a biologically plausible intervention for improving breathing comfort during dental treatments. Available in vitro and volunteer physiological data indicate that the HFNC can generate measurable airway pressure effects and modulate breathing patterns. The magnitude of these effects is influenced by mouth opening/closure, an issue directly relevant to dental treatment where sustained mouth opening is often unavoidable [8]. Physiological studies in patients with spontaneous breathing further support the concept that the HFNC can improve breathing efficiency, consistent with the upper-airway dead-space washout mechanism [9].

Despite their increasing use in other clinical settings, the feasibility of using HFNCs during routine dental treatments has not been adequately investigated, and patient tolerance of HFNCs during dental procedures and the practicality of using HFNCs in dental clinics are yet to be determined. Likewise, the feasibility of collecting physiological and patient-reported outcome data in this setting is uncertain. Although a recent scoping review summarized the application of HFNCs during dental procedures with sedation, the evidence base remains limited. Available evidence is primarily related to oxygenation/ventilatory endpoints under sedation. Importantly, the feasibility of HFNC use in routine (non-sedated) dental treatment has not been sufficiently investigated [10].

Feasibility-related uncertainties must be addressed before conducting a fully powered effectiveness trial. The primary objective of this pilot study is to assess the feasibility of a definitive clinical trial to evaluate the use of a HFNC during dental treatment. The secondary objectives are to explore preliminary patient-centered outcomes related to stress and comfort to inform outcome selection, study procedures, and sample size estimation for a future clinical trial.

## 2. Methods

### 2.1. Study Design

This is an investigator-initiated, single-center, open-label pilot feasibility study that will be conducted at Nagasaki University Hospital, Japan. The study was designed in accordance with the Consolidated Standards of CONSORT extension for pilot and feasibility trials and the principles of the Declaration of Helsinki. Ethical approval was obtained from the Clinical Research Review Board of Nagasaki University, and the study is registered with the Japan Registry of Clinical Trials (jRCTs072240099). Given the exploratory nature of the research question and the need to evaluate feasibility-related parameters before a definitive trial, a pre–post, fixed-sequence, within-subject feasibility design was adopted.

**Figure 1:**
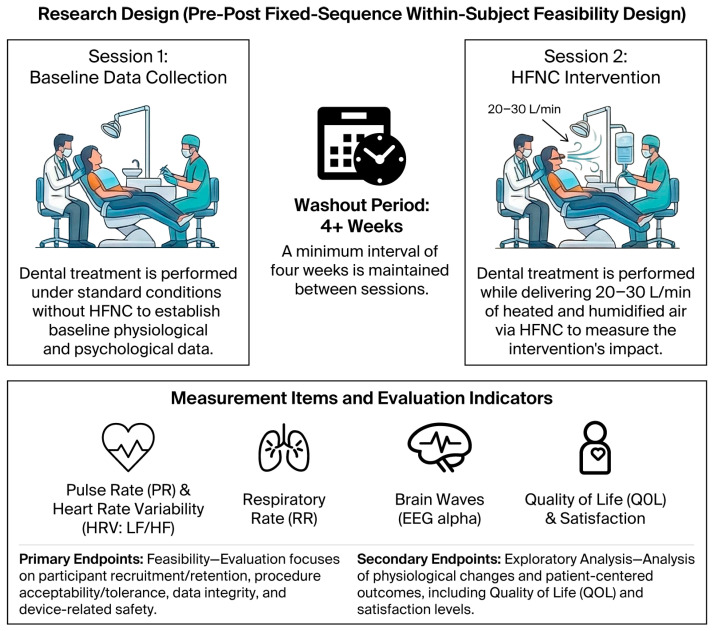
Framework of the pre–post, fixed-sequence, within-subject feasibility design study.

As illustrated in Figure 1, this pilot study was specifically designed as a feasibility-oriented, pre–post, fixed-sequence, within-subject study to evaluate the practicality, acceptability, and safety of high-flow nasal cannula (HFNC) use during routine full-mouth periodontal treatment, rather than to assess clinical effectiveness. Participants will undergo two dental treatment sessions, one without a HFNC and one with a HFNC, separated by a washout period of at least four weeks.

The research design, measurement items, and evaluation indicators for high-flow nasal cannula (HFNC) intervention in full-mouth periodontal treatment are shown in Figure 1, which illustrates the structured approach to evaluating the impact of a HFNC during full-mouth periodontal treatment in a fixed-sequence, pre–post, within-subject feasibility design.

Research design (Pre–Post, Fixed-Sequence, Within-Subject Feasibility Design): The study will be conducted in two sessions separated by a washout period of 4 weeks.

Session 1 (Baseline Data Collection): Dental treatment will be performed under standard conditions without a HFNC to establish baseline physiological and psychological data.

Session 2 (HFNC Intervention): Dental treatment will be performed while delivering 20–30 L/min of heated and humidified air via a HFNC to measure the intervention’s impact.

Measurement items: Data collected include physiological indicators such as Pulse Rate (PR), Heart Rate Variability (HRV: LF/HF), Respiratory Rate (RR), and Brain Waves (EEG alpha), as well as patient-centered metrics like Quality of Life (QOL) and Satisfaction.

The primary outcomes are feasibility parameters, including recruitment and retention rates, patient acceptability and tolerance of the HFNC, completeness of physiological and questionnaire-based data collection, and device-related adverse events. The secondary outcomes are exploratory and include physiological and patient-centered measures to inform outcome selection and sample size estimation for a future definitive clinical trial.

This design allows each participant to serve as their own control and enables comprehensive assessment of feasibility outcomes, including patient tolerance, data completeness, and device-related safety, under real-world dental treatment conditions.

### 2.2. Participants

Adult patients (aged ≥ 18 years) undergoing routine full-mouth periodontal treatment at the Department of Special Care Dentistry, Nagasaki University Hospital, will be eligible for inclusion. Written informed consent will be obtained from all participants prior to enrolment.

### 2.3. Inclusion Criteria

- Patients visiting the Nagasaki University Hospital Dental Department for regular management and scheduled to undergo full-mouth periodontal disease treatment (periodontal pocket cleaning);
- Age ≥ 18 years at the time of consent;
- Ability to understand the study procedures and complete questionnaires independently.

### 2.4. Exclusion Criteria

- Inability to breathe through the nose;
- Cognitive impairment precluding questionnaire completion;
- Pregnancy;
- Any condition deemed unsuitable by the principal investigator.

### 2.5. Study Procedures

After providing informed consent, participants will undergo full-mouth periodontal treatment (approximately less than 30 min in the supine position without a rubber dam under High-Capacity Dental Suction conditions) in two different sessions. During the first session, full-mouth periodontal treatment will be performed without the use of a nasal cannula or other respiratory devices. Participants will subsequently complete a self-administered questionnaire. In the pre–post, fixed-sequence, within-subject feasibility design, stress during the second session may decrease independently of the HFNC due to factors such as habituation, reduced treatment volume/invasiveness, and changes in psychological adaptation. Therefore, the following points will be carefully considered. The two sessions will be conducted and standardized at the same time of day, by the same operator with five years of clinical experience using same instrumentation type with consistent treatment extent and duration, and each session involves the treatment of only one side of the jaw or one quadrant within the full-mouth periodontal treatment area in a total of two sessions. In addition, to minimize the possibility of marked changes in periodontal status by the second session, the interval between the two sessions will be limited to within four weeks.

The second full-mouth periodontal treatment session will be scheduled for at least four weeks after the first session. During this session, full-mouth periodontal treatment will be performed with a dedicated HFNC (AIRVO3, Fisher & Paykel Healthcare Ltd., Auckland, New Zealand). Prior to the start of treatment, the nasal cannula will be inserted into both nostrils, and warmed and humidified air will be delivered at an initial flow rate of 20 L/min. If no discomfort is reported, the flow rate will be gradually increased to a maximum of 30 L/min. The AIRVO3 HFNC will be applied with fixed FiO_2_ at 0.21 at a temperature of 37 °C, with appropriate matched cannula size (S, M, L size) for all patients and criteria for flow escalation and discontinuation.

During both sessions, physiological parameters will be continuously monitored, including pulse rate, respiratory rate, and electrocardiographic RR intervals. Autonomic nervous system activity will be assessed using heart rate variability analysis (LF/HF ratio) using an MWM20 ECG monitor (GMSCo., Ltd., Tokyo, Japan) (sampling rate 200 Hz, 30 s window, placing ECG electrode on the anterior chest and behind the ears). For the ECG analysis, RR intervals will be resampled at 1000 Hz. The pulse rate and respiratory rate will be summarized as mean intra-procedural values and the value of LF/HF will be calculated over a fixed 2 min segment. The baseline measurement is defined as a value before the start of high-flow therapy, percent changes against baseline value will be derived for change score, and the missing or low-quality signal segments will be carefully checked with existence of large artifact or noise signals.

Stress-related electroencephalographic activity will be evaluated by assessing alpha wave appearance rates using the MWM20 EEG monitor (sampling rate 200 Hz, 2 s window, placing EEG electrode on the forehead and behind the ears). Following treatment, participants will complete a questionnaire assessing comfort and anxiety. Because electroencephalography (EEG) signals are susceptible to artifacts, particularly from electromyographic activity caused by blinking and eye movements, preparatory measures will be taken to minimize artifact contamination. During measurement, participants will be required to keep their eyes closed and remain in an environment designed to prevent body movement, and recordings will be performed in an environment designed to prevent body movement. During electrocardiography (ECG), RR intervals will be analyzed; therefore, automatic processing for arrhythmia detection and noise removal will be applied. A zero-phase filter and a hum filter will be used as baseline settings. EEG recordings will be obtained using a monopolar derivation with a reference electrode placed behind the ear.

No waveform correction will be performed during measurements using either the MWM20 EEG or ECG monitor. In the dental treatment setting, swallowing, mandibular movements, instrument noise, and body motion may also further interfere with EEG α-wave recordings. Therefore, the protocol restricts measurements to standardized short resting periods (an approximately two-minute resting state) for stable measurement before and after treatment rather than during instrumentation itself. It should be noted that the minimum valid recording length required for EEG α-wave analysis will be maintained for at least two minutes, with no large spikes in waves. All the participants must avoid swallowing saliva, opening or closing the mouth, and body movement during the measurement. In EEG (electroencephalogram) data, sections with noise may show large spikes in delta waves. In EEG data, sections with noise may show large spikes in EEG waves.

This is primarily believed to be caused by significant electromyographic activity, such as body movements or blinking. In MW2, data is output using 4 s windows shifted by 2 s.

This is then averaged over 10 s and exported as a CSV file. Since the numerical values in noisy sections change significantly for only an instant, removing those sections afterward serves as a method to address noise and artifacts. The EEG α-wave activity will be expressed as a mean appearance rate during a predefined resting window. In this feasibility study, the evaluation of EEG α-wave analysis would be a highly exploratory feasible measure.

The baseline value will be established prior to the intervention, and signal analysis will be performed using automated analysis with MemCalc™.

The protocol will be stopped if a significant risk to the research subjects or extreme discomfort is identified. For example, if patients experience adverse events such as epistaxis, discomfort, aspiration/cough events, or desaturation according to vital sign monitoring, the principal investigator should adjudicate the AEs and should stop the protocol.

In this study, safety and infection control in the dental operatory require explicit handling. HFNC therapy introduces airflow, humidification, and interface equipment in a setting with aerosols and cross-infection risk. We will use a single-use circuit and cannula with a built-in filter device. After completion of each session, the AIRVO3 machine will be decontaminated using a special cleaning circuit.

As with conventional hypoxic therapy interventions, there are concerns about the occurrence of adverse events—such as a decrease in SpO_2_—for which a causal relationship cannot be ruled out. According to past reports, the majority of these adverse events are mild to moderate in severity, and studies have shown that they can be rapidly resolved through airway management techniques and control of the depth of sedation. The AIRVO3 device allows for confirmation of SpO_2_ sensor attachment and respiratory rate during monitoring, and an alarm can be set to sound when the SpO_2_ level falls below 90%.

The principal investigator (PI), as the person responsible for evaluation, will ensure that appropriate interventions are implemented for any adverse events of concern. It has been noted that high-flow nasal cannulation may generate aerosols when the flow rate exceeds the patient’s tidal volume, and the use of surgical masks, goggles, and rubber gloves is mandatory during the procedure to prevent operator infection. These measures have been proven effective in preventing aerosol-transmitted infection during the COVID-19 pandemic.

A questionnaire survey will score dental treatment comfort using a 5-point scale across 9 items (1: Not at all, 2: Slightly, 3: Somewhat, 4: Quite a lot, 5: Very much). (File S1)

Q1: Did your body tense up during treatment?
Q2: Did your breathing become faster during treatment?
Q3: Did you sweat during treatment?
Q4: Did you feel nauseous or experience vomiting during treatment?
Q5: Did your heartbeat quicken during treatment?
Q6: Was it easy to breathe through your nose during treatment?
Q7: Were you able to swallow saliva or water residue effectively during treatment?
Q8: Did you feel anxious during treatment?
Q9: If you were to use it again, which method would you prefer: the first or second?

For Q1~Q8, the Likert scale will be administered in numerical order.

Q9 (Nominal scale: preference) will be administered only after the second session and will be used as a distinct nominal item.

### 2.6. Outcomes (Table 1)

#### 2.6.1. Primary Feasibility Outcomes

The primary feasibility outcomes are as follows:

- Recruitment ≥ 5/month (30 cases/6 months);
- Retention rates ≥ 70%;
- Patient tolerance and acceptability of the HFNC during dental treatment ≥ 80%;
- Completeness of physiological and questionnaire-based data collection ≥ 80%;
- Occurrence of device-related adverse events at less than 5% (less than one case).

**Table 1:** Outcomes table.

Category	Outcome	Purpose
Primary (Feasibility)	Recruitment, retention	Trial feasibility
	Acceptability, tolerance	Practical implementation
	Data completeness	Outcome feasibility
	Adverse events	Safety
Secondary (Exploratory)	Pulse rate, respiratory rate	Stress physiology
	HRV (LF/HF)	Autonomic response
	EEG alpha activity	Neurophysiological stress
	Questionnaire	Patient-centered outcomes

#### 2.6.2. Secondary Exploratory Outcomes

Secondary outcomes will be exploratory and include the following:

- Pulse rate and respiratory rate during treatment;
- Autonomic nervous system activity assessed via RR interval analysis;
- Stress-related electroencephalographic alpha wave activity;
- Patient-reported comfort and anxiety assessed using a nine-item questionnaire.

#### 2.6.3. Statistical Analysis

As this is a pilot feasibility study, formal hypothesis testing is not planned. Feasibility and exploratory outcomes will be summarized descriptively. Analyses of secondary outcomes will be exploratory and intended to inform the design of future definitive trials. Regarding continuous outcomes, we will estimate the mean difference between treatment with a HFNC and treatment without a HFNC, along with its 95% confidence interval.

In the case of missing data, the proportion of missing values will be reported for each variable at each time point. Summary statistics will be calculated excluding missing values. Differences between time points will be assessed after excluding subjects with missing values, as this study will include only two time points.

#### 2.6.4. Sample Size

As this is a pilot feasibility study, a formal sample size calculation based on statistical power was not performed. Approximately 30 patients per year who meet the eligibility criteria are treated at the Department of Special Care Dentistry of Nagasaki University Hospital. Therefore, a target sample size of 30 participants was considered feasible within the planned study period and sufficient to assess feasibility outcomes, including recruitment, acceptability, data completeness, and safety, to inform the design of future definitive trials.

## 3. Discussion

The aim of this pilot feasibility study is to determine novel and clinically relevant insights relating to the use of a HFNC during routine dental treatment. Dental procedures frequently require prolonged mouth opening with spontaneous nasal breathing. Patient posture can also compromise nasal airflow and hinder saliva swallowing. These factors can contribute to discomfort, anxiety, and stress. Currently, there are limited evidence-based strategies specifically designed to address the common physiological and psychological challenges in dental settings. Importantly, certain dental procedures (e.g., rubber dam isolation) are known to increase discomfort and treatment distress due to swallowing or breathing difficulties [11].

The physiological mechanisms underpinning the potential utility of HFNCs in dental settings warrant careful consideration, particularly because dental procedures impose unique respiratory challenges that differ from those in the perioperative or critical care contexts.

**Figure 2:**
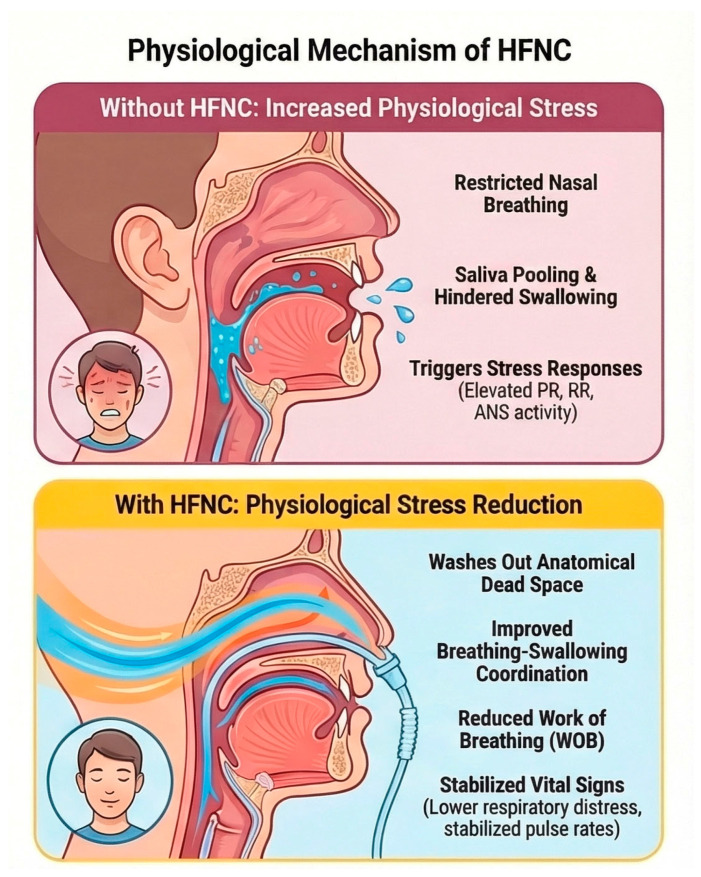
Proposed physiological mechanisms by which a high-flow nasal cannula (HFNC) may influence breathing comfort during dental treatment. During routine dental procedures requiring sustained mouth opening, nasal airflow limitation, impaired breathing–swallowing coordination, and increased work of breathing may contribute to physiological stress responses. The HFNC delivers warmed and humidified high-flow air, which may stabilize nasal airflow, facilitate nasopharyngeal dead-space washout, and improve breathing–swallowing coordination under spontaneous breathing conditions. These mechanisms provide a biological rationale for investigating the HFNC as a patient-centered respiratory support strategy in the dental setting.

Figure 2 provides a conceptual framework summarizing the proposed physiological mechanisms by which HFNC therapy may influence breathing comfort during dental treatment, particularly under conditions of sustained mouth opening and spontaneous breathing. During routine dental procedures, prolonged mouth opening may lead to nasal airflow limitation, impaired breathing–swallowing coordination, and increased breathing work, which can contribute to physiological stress responses.

These conditions may amplify patient concerns, such as anxiety, choking, or difficulty swallowing saliva, further contributing to dental fear (in both adult and pediatric populations) [10].

By delivering warmed and humidified high-flow air, the HFNC may stabilize nasal airflow, facilitate nasopharyngeal dead-space washout, and improve breathing–swallowing coordination, thereby reducing respiratory effort and stress. These mechanisms contribute to the biological rationale for investigation, rather than being evidence of clinical effectiveness.

Additionally, the HFNC may improve the coordination between breathing and swallowing [3,4,5,6,7,12], a function that is critically challenged during dental procedures. A recent scoping review highlighted the conflicting results of studies that have explored the effects of the HFNC on swallowing function and swallowing–breathing coordination, confirming the need for context-specific feasibility studies before definitive effectiveness trials are undertaken [6]. These considerations further support the rationale for evaluating the HFNC under dental-specific conditions, rather than extrapolating findings from perioperative or critical care settings [1]. The primary objective of this pilot study is not to establish the clinical effectiveness of the HFNC in reducing stress but to determine whether its use during dental treatment might be feasible, acceptable to patients, and compatible with routine dental workflows. The assessed feasibility outcomes, including patient tolerance, acceptability, data completeness, and device-related safety, are essential prerequisites for the successful design and execution of future definitive clinical trials. Importantly, this study intends to demonstrate that HFNCs can be used in dental clinics and that comprehensive physiological and patient-reported data can be collected without disrupting standard dental procedures.

A distinctive strength of this study is its multidimensional assessment of stress-related outcomes by incorporating physiological parameters, autonomic nervous system indices, electroencephalographic activity, and patient-reported measures. Although these outcomes are explicitly exploratory, their inclusion offers valuable preliminary information on the feasibility and relevance of different outcome domains in dental settings. This approach is supported by recent systematic reviews on dental anxiety that emphasized substantial heterogeneity in outcome measures and highlighted the need for pragmatic, patient-centered endpoints that can be collected in routine clinical settings [10]. Concerns related to choking, gagging, or difficulty in managing saliva during treatment further reinforces the clinical relevance of interventions that can stabilize breathing and support breathing–swallowing coordination. Swallowing safety should be considered when applying HFNC to new clinical contexts, given that aspiration events have been reported during instrumented swallowing evaluations in certain clinical populations employing HFNCs [13].

Dental anxiety and treatment-related distress remain common clinical problems. Contemporary evidence highlights persistent gaps and heterogeneity in interventions aimed at reducing acute anxiety during dental procedures, underscoring the need for pragmatic, low-burden approaches that are easily integrated into routine workflow [1,14,15,16]. Such conditions may also amplify patient anxiety related to concerns about choking or an inability to swallow saliva effectively, which have been identified as important components of dental fear in both adult and pediatric populations [15,16].

### Limitations

This study has several limitations. First, it is a single-center, open-label, pilot feasibility study, and the findings are not intended to be generalizable or establish clinical efficacy. The exploratory analyses are not powered for hypothesis testing. Therefore, the results should be interpreted cautiously. However, these characteristics are inherent in pilot feasibility studies and are appropriate, given the primary objective of informing future study designs.

Second, the target sample size was determined pragmatically based on the annual number of eligible patients at the study institution and the anticipated consent rates, rather than on formal power calculations. Nevertheless, the comprehensive collection of physiological (EEG and ECG) and patient-reported data provides valuable preliminary information to guide the outcome selection and sample size estimation for subsequent confirmatory studies.

Third, the open-label, pre–post, fixed-sequence, within-subject feasibility design may be susceptible to expectation and sequence effects despite the inclusion of a washout period. In addition, stress and comfort will partly be assessed using self-reported questionnaires, which may be influenced by prior experience or recall bias.

Fourth, this study will use a fixed-sequence, within-subject comparison with the first full-mouth periodontal treatment without a HFNC, followed by a second session with a HFNC to minimize patient anxiety regarding HFNC use. Informed consent will be obtained before the interventions. However, a randomized crossover study design would be more appropriate for the objective evaluation of outcomes, as such a design could minimize the influence of period effects, learning or conditioning effects, and expectation bias, particularly when subjective endpoints such as comfort and anxiety are assessed. In the future, randomized crossover designs should be further examined.

Finally, the study will involve only patients undergoing full-mouth periodontal treatment (each session will be 30 min or less, with the patient in the supine position and no rubber dam). Future studies investigating the use of a HFNC during other dental procedures, such as extractions or endodontic therapy, are warranted. Furthermore, caution will be required when extrapolating these findings to other dental treatment contexts.

## 4. Conclusions

This RCT study is designed to assess the feasibility, acceptability, safety, and preliminary physiological effects of HFNC during periodontal treatment. By focusing on feasibility-related outcomes and exploratory patient-centered measures, this pilot study provides a methodological and physiological rationale for conducting a fully powered clinical trial to evaluate the effectiveness of the HFNC for improving breathing comfort and reducing treatment-related stress in routine dentistry in future-oriented studies.

These findings may suggest that delivering warmed and humidified high-flow air may address common sources of discomfort during dental treatment, particularly difficulties related to nasal breathing and swallowing saliva, which may contribute to dental anxiety. Although no conclusions regarding clinical effectiveness can be drawn from this exploratory pilot study, the results support subsequent confirmatory trials.

Ultimately, further research informed by these feasibility data has the potential to establish the HFNC as a novel, low-burden, patient-centered adjunct to routine dental care, with implications for improving treatment tolerance, safety, and overall oral health outcomes.

## Data Availability

The original contributions presented in this study are included in the article/Supplementary Materials. Further inquiries can be directed to the corresponding author.

## Supplementary Materials

The following supporting information can be downloaded at https://www.mdpi.com/article/10.3390/dj14040208/s1: File S1: questionnaire-based data collection.
