# Supplementary material for: Feasibility and Safety of High-Flow Nasal Cannula Use During Dental Treatment: A Pilot Study
Source: Dent J (Basel). 2026 Apr 2;14(4):208. doi: 10.3390/dj14040208 (PMC13115242; doi:10.3390/dj14040208)
Supplement: Supplementary file 1 [file dentistry-14-00208-s001.zip › dentistry-4122991-supplementary.pdf]

questionnaire-based data collection

The comfort of dental treatment (evaluated by nine questions) between the first and second visits.
Please evaluate the following questions on a five-point scale.
1: None at all, 2: A little, 3: Somewhat, 4: Quite a bit, 5: Very much
1: None at all, 2: Slightly, 3: Somewhat, 4: Quite a bit, 5: Very much

Q1: Did your body feel stiff during treatment?
Q2: Did your breathing become faster during treatment?
Q3: Did you sweat during treatment?
Q4: Did you feel nauseous or experience vomiting during treatment?
Q5: Did your heartbeat become faster during treatment?
Q6: During treatment, was it easy to breathe through your nose?
Q7: During treatment, were you able to swallow saliva or water effectively?
Q8: During treatment, did you feel anxious?
Q9: If you were to use it again, which method would you prefer—the first or second? (Administered only after the second session)
